# Positive Selection and Duplication of Bat TRIM Family Proteins

**DOI:** 10.3390/v15040875

**Published:** 2023-03-29

**Authors:** Jiazheng Xie, Bowen Tan, Yi Zhang

**Affiliations:** Chongqing Key Laboratory of Big Data for Bio Intelligence, Chongqing University of Posts and Telecommunications, Chongqing 400065, China

**Keywords:** Chiroptera, TRIM, antiviral immunity, positive selection, gene duplication

## Abstract

Bats have received increasing attention because of some unique biological features they possess. TRIM is a large family of proteins that participate in diverse cellular functions, such as antiviral immunity, DNA damage repair, tumor suppression, and aging. These functional areas appear to be highly consistent with the special characteristics of bats, such as tolerance to viruses and DNA damage generated in flight, low cancer incidence, and longevity. However, there is still a lack of systematic study of the TRIM family in bats. Here, we explored the TRIM family of bats using the genomes of 16 representative species. The results showed that the bat TRIM family contains 70 members, with 24 under positive selection and 7 duplicated. Additional transcriptomic analysis revealed the tissue-specific expressions of *TRIM9*, *46*, *54*, *55*, *63*, and *72*. Additionally, following interferon or viral stimulation, *TRIM* orthologs associated with antiviral immunity reported in humans were also upregulated in bat cells. The present study systematically analyzed the composition, evolution, and expression of bat *TRIM* genes. It may provide a theoretical basis for studies of bat TRIM in the fields of antiviral immunity, longevity, and tolerance to DNA damage.

## 1. Introduction

Bats (Chiroptera) are the second most species-rich and abundant group of mammals and can be subdivided into two suborders, Yangochiroptera and Yinpterochiroptera. They have been in the spotlight recently for some peculiarities, such as being the only mammals capable of sustained flight, laryngeal echolocation, longevity, and reservoirs of viruses. More than 200 viruses from over 27 families have been detected in bat samples. Many of these are fatal to humans, such as the Ebola virus, lyssavirus, SARS coronavirus, and Henipavirus [1]. In recent years, there have been recurring spillover events of viruses carried by bats that caused infections in humans or livestock. For example, the bat-derived SARS coronavirus caused more than 8000 infections worldwide in 2003, with a mortality rate of approximately 10% [2]. In 2016–2017, a new bat-derived coronavirus, swine acute diarrhea syndrome coronavirus (SADS-CoV), caused the death of more than 20,000 piglets [3]. Additionally, bats are also the most likely reservoir of the emerging coronavirus, SARS-CoV-2 [4]. Thus, the question arises as to why bats can harbor so many viruses. One speculation is that bats’ immune systems may have adapted to tolerate viruses [5].

However, research on the bat immune system is still in its infancy, with multiple hurdles, including the scarcity of bat research samples, tools, and experiment model systems [6]. However, with the development of high-throughput sequencing technology, more and more bat genome and transcriptome data are being released. Bat1K, an initiative aimed at sequencing the genomes of all living bat species, will promote an understanding of unique bat adaptations [7]. Furthermore, there are growing comparative studies related to bat antiviral immunity, such as the discoveries of constitutively expressed IFN-α, dampened STING-dependent interferon response and NLRP3 inflammasome activation in bats [8,9,10]. However, studies related to the regulation of immune response in bats remain rare.

The TRIM is a large protein family that plays important roles in the regulation of immune response, as well as in biological processes such as autophagy, carcinogenesis, cell cycle, and DNA damage repair. They consist of at least three domains: RING, B-box, and coiled-coil domain (CCD). Besides these domains, the C-terminus of TRIM proteins may contain some specific domains, such as PRY/SPRY (B30.2), PHDs, and NHL [11,12]. Most of the TRIM family members are E3 ubiquitin ligases. The N-terminal cysteine-rich RING domain can catalyze the ubiquitin or ubiquitin-like (UBL) modification of the target proteins to regulate their stability or activation state [13]. For example, TRIM25 catalyzes the K63-linked polyubiquitination of RIG-I, which promotes RIG-I multimerization and is essential for downstream pathway activation [14]. Additionally, TRIM21, also known as Ro52, catalyzes K48-linked ubiquitination and the degradation of IRF3 to negatively regulate interferon production and to avoid autoimmune diseases caused by excessive immune responses [15].

In addition to regulating the antiviral immune response, some TRIM members can directly inhibit viral replication. For example, TRIM22 can inhibit the budding of virus-like particles by disrupting the proper trafficking of the virus structural protein Gag [16]. Additionally, TRIM5α can block some retroviruses by accelerating the uncoating of retroviral capsids [17]. Interestingly, species-specific variation of TRIM5α can influence the ability to restrict HIV. For instance, rhesus monkey TRIM5α has greater potency to block HIV-1 infection than human TRIM5α [18].

The number of TRIM family members varies among species. Whereas humans have 73 *TRIM* genes, fruit flies have only 7, and most jawed fish species have more than 100 *TRIM* genes [19]. A study aimed at identifying candidate antiviral restriction factors in the human TRIM family showed that 17 *TRIM* genes in humans are under positive selection [20]. In bats, a recent study of the *TRIM6/34/5/22* gene cluster showed positive selection and duplication events happened in *TRIM5/22* [21]. However, no study has been performed to examine other bat TRIM family members. Here, we conducted a genome-wide identification, evolution, and expression analysis of the bat TRIM family for the first time, and the results will be helpful for further functional studies of these genes.

## 2. Materials and Methods

### 2.1. Bat Genome Data Retrieval

In this study, RefSeq genomes of 16 representative species of bat were downloaded from NCBI (https://www.ncbi.nlm.nih.gov/data-hub/genome/?taxon=9397 accessed on 5 January 2021) and analyzed (Table 1).

### 2.2. Identification of TRIM Proteins from Bats

To identify putative TRIM proteins in bats, profiles of conserved RING (RING_PF13639) and B-box (B-box_PF00643) domains were retrieved from Pfam [22] and used to perform an HMM search [23] against all predicted proteins of bats listed in Table 1, with an E-value cutoff of 1 × 10^−3^. As RING and B-box domains are not unique to TRIM proteins, all candidates identified were further verified using blast [24] against well-annotated human and mouse proteins to exclude non-TRIM proteins. The final TRIM proteins are listed in (Table S1).

### 2.3. Phylogeny and Motif Analysis

TRIM family proteins of *Myotis lucifugus* were aligned with MAFFT with -maxiterate 1000 [25]. Next, a maximum-likelihood tree with a JTT + CAT model was inferred by FastTree with default parameters [26]. Additionally, MEME [27] was used to identify conserved motifs with the following parameters: 15 as the maximum number of motifs, and the motif width between 6 and 250 residues.

### 2.4. Positive Selection Analysis

*TRIM* genes from the 16 representative species of bats were analyzed with CODEML from the PAML package v.4.9 [28]. The site model was used to detect putative positive selection with a nonsynonymous/synonymous ratio (also called ω) >1 in bat *TRIM* genes. The likelihood ratio (LTR) test method was introduced to compare the neutral model M7 (ω varies according to the beta distribution and restrict ω ≤ 1) versus alternative model M8 (beta distribution and allow ω > 1). Additionally, the Bayes Empirical Bayes (BEB) approach was applied to infer posterior probability (pb), and only the site with ω > 1 appeared in the LRT test, and pb > 0.90 was considered as a positively selected site. We also estimated ω through HYPHY (v 2.3.14) by the SLAC, FEL, MEME, and FUBAR methods [29].

### 2.5. RNA-Seq Data Analysis

Bat transcriptome data were aligned to the bat genome by STAR aligner (v2.7.10b) [30] with the default parameters, except for the transcriptomic analysis of *Myotis daubentoniid*, which lacks a reference genome. In the transcriptomic analysis of *Myotis daubentoniid*, reads were mapped to the reference genomes of *Myotis lucifugus* with adjusted —outFilterScoreMinOverLread and —outFilterMatchNminOverLread of 0.4. Normalized gene expression values were calculated by featureCounts [31] as RPKM. DESeq2 was employed to calculate the fold-change (FC) and *p*-value of the mapped genes [32]. Only genes with an FC ≥ 2 and an adjusted *p*-value < 0.05 were defined as differentially expressed genes.

## 3. Results

### 3.1. Number of Bat TRIM Family Members

**Figure 1:**
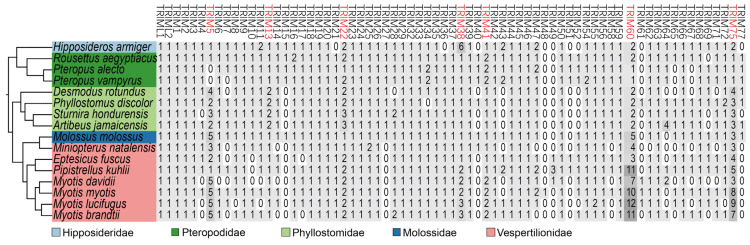
Number of bat TRIM family members. The bat phylogeny tree was generated using data from a previous study [33]. The background color is proportional to the corresponding copy number. The duplicated TRIM family members are marked in red.

A total of 70 TRIM family members were identified in 16 representative species of bats (Figure 1). In agreement with a previous study, bat *TRIM5* and *TRIM22* were duplicated [21]. In addition, we found that *TRIM13*, *38*, *41*, *60*, and *75* in bats also undergo gene duplication. Interestingly, the copy number of *TRIM60* and *TRIM75* greatly expanded in Yangochiroptera, with 12 copies of *TRIM60* and 9 copies of *TRIM75* in *Myotis lucifugus*, for example. To better understand the extraordinary expansion of *TRIM60* and *TRIM75*, we examined the *TRIM60* and *TRIM75* locus in bats and other mammals. The genomic diagram (Figure S1) showed that *TRIM60*, *TRIM61*, and *TRIM75* are clustered, with *TMEM192* and *APELA* as the boundary. Although the gene copies of bat *TRIM60/61/75* increased, the total length of the cluster did not significantly increase.

### 3.2. Phylogeny and Motif Distribution of Bat TRIM Family Proteins

**Figure 2:**
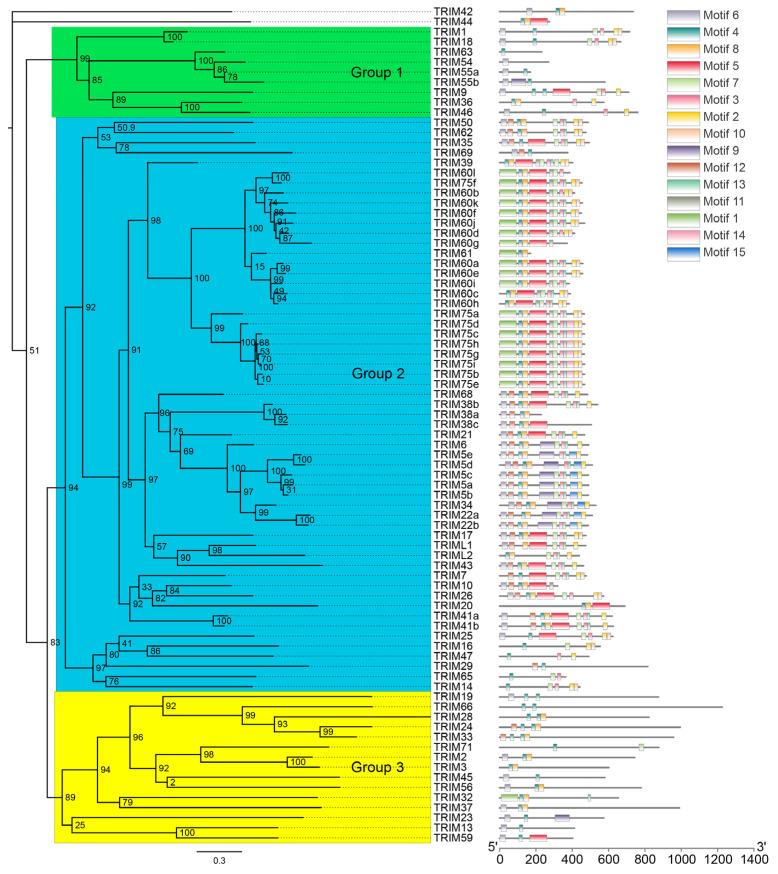
Phylogeny and motif composition of TRIM family proteins of *Myotis lucifugus*. The phylogeny tree was constructed by FastTree, and the motif composition was analyzed by MEME.

To gain insights into the diversity of bat TRIM family members, TRIM proteins of *Myotis lucifugus* were aligned using MAFFT, a maximum-likelihood phylogenetic tree was created by FastTree, and the conserved motifs were predicted via MEME (Figure 2). The bat TRIM family proteins can be classified into three groups, with most members in group 2. Interestingly, the duplicated *TRIM5*, *22*, *38*, *41*, *60*, and *75* are all located in group 2. Moreover, members in group 2 have more conserved motifs. The tendency of the duplication and generation of motifs may underlie their novel functions. These observations are consistent with a previous study of the TRIM family in humans that showed that the group 2 genes are younger, highly dynamic, and might be a source of novel TRIM functions [34].

### 3.3. Positive Selection Analysis of Bat TRIM Family Genes

Positive selection promotes advantageous variants’ fixation in a population. To determine *TRIM* orthologous genes under positive selection in the 16 bat species, PAML and HYPHY were applied using a *p*-value cutoff of 0.05 or a posterior probabilities cutoff of 0.9. We identified 24 out of 70 bat *TRIM* genes as having significant evidence of positive selection supported by both PAML and HYPHY (Table 2 and Table S2). Consistent with the previous report, *TRIM5* and *TRIM22* were under positive selection in bats [21]. Furthermore, another 22 *TRIM* genes were also subjected to positive selection. TRIM21 can bind antibodies and target antibody-bound viruses to proteasome degradation [35]. The positive selection of bat TRIM21 may be related to the highly diverse antibody repertoire [36] and may empower bats to respond rapidly to emerging viruses.

### 3.4. Tissue Expression Analysis of Bat TRIM Genes

To further understand the function of these duplicated bats’ *TRIM* genes, we analyzed their tissue expression level using public transcriptomic data (SRR11528215–SRR11528221) [37] of *Myotis myotis*, a Yangochiroptera bat in which most *TRIM* duplication events occurred.

**Figure 3:**
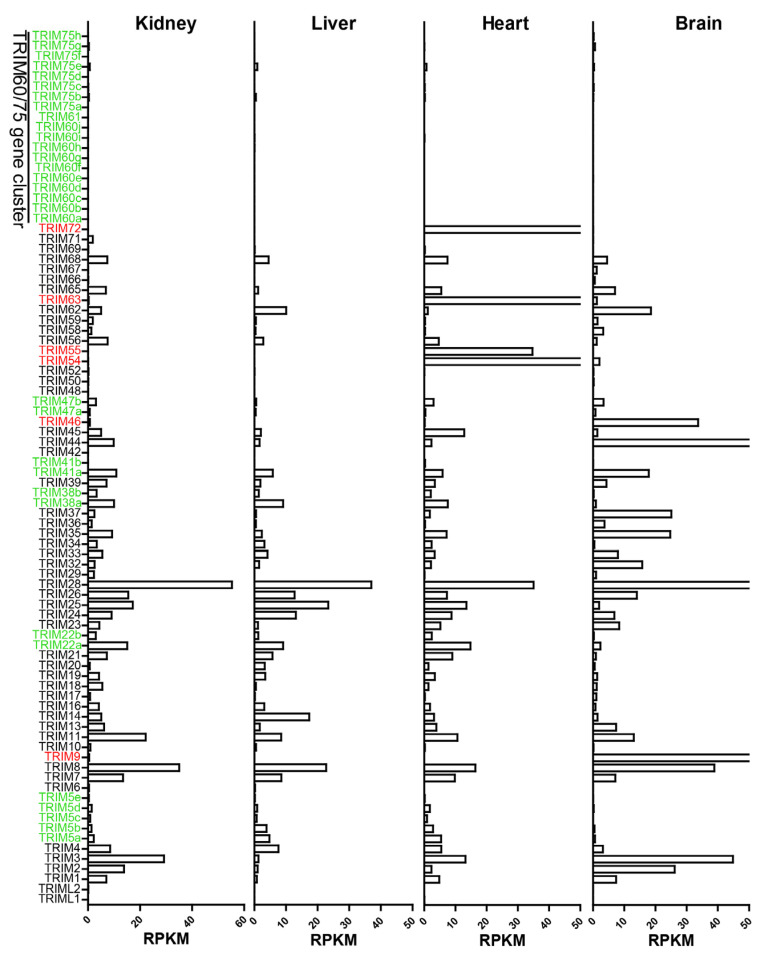
RPKM of bat *TRIM* genes in transcriptome data from *Myotis myoti* tissues. The duplicated genes are marked in green, and the tissue-specific expressed genes are marked in red.

We calculated the RPKM of *TRIM* genes in *Myotis myoti* tissues: kidney, liver, heart, and brain. The results (Figure 3) showed that duplicated bat *TRIM* paralogs have considerable expression, except for *TRIM60/75.* However, the expression levels of duplicated *TRIM5*, *22*, and *38* are clearly lower in the brain than in other tissues. Interestingly, *TRIM54*, *55*, *63*, and *72* are primarily expressed in the heart, and *TRIM9* and *46* in the brain. This phenomenon is consistent with their striated muscle or neuron-specific functions [38,39,40]. It is worth noting that previous research showed that heart-specific expression of *TRIM72* varies considerably in primates, with higher levels of expression in high-heart-rate species. Additionally, its expression can affect mitochondrial respiration by altering the genes involved in oxidative phosphorylation [41]. Thus, the heart-specific expression of bat *TRIM72* may reflect the increasing energy metabolism demand during powered flight.

### 3.5. Bat TRIM Genes Regulated by Virus Infection or Type I IFN Treatment

To better understand the antiviral function of bat *TRIM* genes, we also analyzed the differential *TRIM* gene expression of cells from two Yangochiroptera bats, *Myotis lucifugus* [42] and *Myotis daubentonii* [43], that were stimulated with IFN alpha or infected with rift valley fever virus (RVFV) (Table 3).

**Figure 4:**
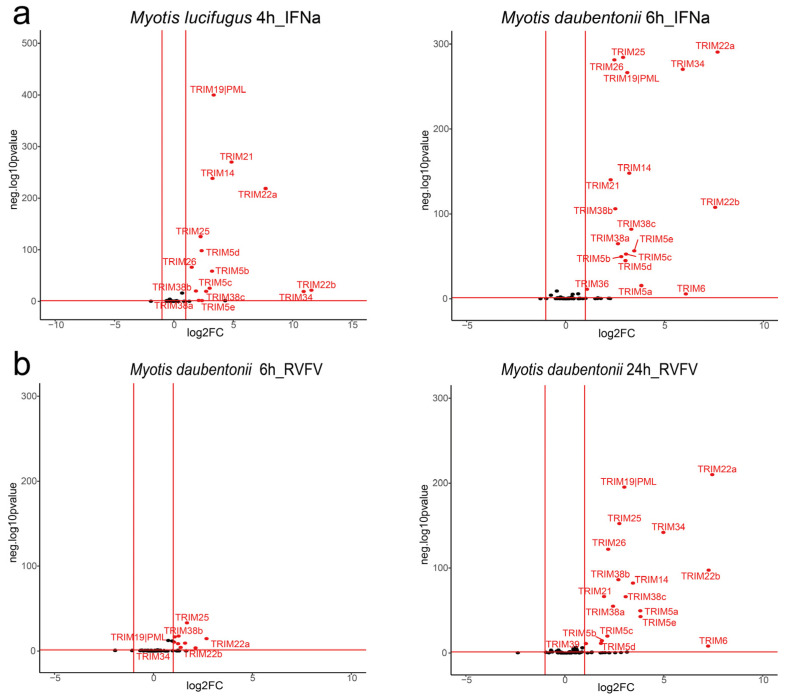
Differential *TRIM* gene expression of bat cells stimulated with IFN alpha (**a**) or infected with RVFV (**b**). On the *X*-axis is the log2 fold-change, and on the *y*-axis is the −log10 *p*-value. For more clarity, only *TRIM* genes are displayed, and those upregulated by at least two-fold with *p*-value less than 0.05 are highlighted in red.

As shown in (Figure 4), although the stimuli or cell sources differ, the profiles of upregulated *TRIM* genes are almost the same. After being stimulated with IFN alpha or infected with RVFV, the expression levels of *TRIM5*, *6*, *14*, *19*, *21*, *22*, *25*, *26*, *34*, and *38* were upregulated. Notably, duplicated paralogs of *TRIM5*, *22*, and *38* were all upregulated. On the contrary, paralogs of the *TRIM60/75* cluster were not upregulated. It seems that, unlike duplicated *TRIM5*, *22*, and *38*, *TRIM60/75* paralogs were not implicated in the antiviral immune response.

## 4. Discussion

In accordance with their diverse physiological functions, TRIM proteins evolve rapidly and might act as a reservoir for novel genes. For instance, variable copies of novel *TRIM* genes have been identified in different human populations [44]. We hypothesize that arms races between bats and viruses might drive the occurrence of novel functions in bat *TRIM* genes. As expected, we found that duplication events occurred in bat *TRIM5*, *13*, *22*, *38*, *41*, *60*, and *75*; most of these are related to antiviral immunity. Furthermore, 24 bat TRIM members are under positive selection. Duplication and positive selection are two major mechanisms of adaptive evolution [45]. Thus, the duplication and high proportion of *TRIM* genes under positive selection pressure (24/70, 34.3%) in bats may highlight the unique adaptation of their immune system.

In agreement with a previous study on human *TRIM* gene expression [46], many antivirus-related bat *TRIM* orthologs are sensitive to IFN. When stimulated with IFN or infected with viruses, their expression levels are upregulated. Notably, the fold change of duplicated *TRIM22a* and *TRIM22b* is the highest among them. As TRIM22 plays important roles in the inhibition of diverse viruses, such as HIV-1, HCV, EMCV, HBV, and IAV [47], the antiviral function of duplicated bat TRIM22 needs further study.

At first, the significantly duplicated *TRIM60/75* attracted our attention. However, the expression analysis showed that bat *TRIM60/75* paralogs were rarely transcripted in the inspected tissues and did not upregulate after interferon or virus stimulation. Therefore, we concluded that bat *TRIM60/75* paralogs might not be implicated in the antiviral immune response. Although the functions of duplicated *TRIM60/75* remain elusive, we can still make some hypotheses. A recent report on mice showed that Trim60 could suppress proinflammatory cytokine production in macrophages [48]. Another report on TRIM60 showed that it most likely plays a role in the spermatogenesis process and is important for spermatid translocation [49]. In some bats, fertilization does not occur during mating, but sperm is stored in the female’s reproductive tract for several months, and ovulation and fertilization only begin after hibernation [50]. Thus, the duplicated bat *TRIM60/75* may help bats avoid tissue damage caused by an overwhelming immune response or delayed fertilization.

## 5. Conclusions

Due to the scarcity of bat research samples and experiment model systems, bat genome and transcriptome data are invaluable resources for bat research. Here, we used the data from the genomes of bats to identify *TRIM* genes and scanned for genes subject to adaptive molecular evolution. In addition, bat transcriptomic data were used to test *TRIM* gene expression and upregulation after IFN stimulation or virus infection. We showed that duplication and positive selection commonly exist in bat *TRIM* genes, the expression of some members is tissue-specific, and some members with antiviral functions can be upregulated by IFN or virus stimulation. These will further our understanding of the adaptation of the bat immune system.

## Figures and Tables

**Table 1:** Information of bat genome data.

Suborder	Family	Species	Assembly Name	Coverage (×)	Scaffold N50 (kb)
Yinptero- chiroptera	Pteropodidae	*Pteropus vampyrus*	Pvam_2.0	188	5954
		*Pteropus alecto*	ASM32557v1	110	15,955
		*Rousettus aegyptiacus*	mRouAeg1.p	64.6	113,812
	Hipposideridae	*Hipposideros armiger*	ASM189008v1	218.6	2328
Yango- chiroptera	Molossidae	*Molossus molossus*	mMolMol1.p	58.3	110,665
	Vespertilionidae	*Myotis davidii*	ASM32734v1	110	3454
		*Myotis myotis*	mMyoMyo1.p	90.9	94,449
		*Myotis brandtii*	ASM41265v1	120	3226
		*Myotis lucifugus*	Myoluc2.0	7	4293
		*Eptesicus fuscus*	EptFus1.0	84	13,455
		*Pipistrellus kuhlii*	mPipKuh1.p	80.8	80,237
		*Miniopterus natalensis*	Mnat.v1	77.0	4315
	Phyllostomidae	*Phyllostomus discolor*	mPhyDis1.pri.v3	65.8	171,743
		*Sturnira hondurensis*	WHU_Shon_v2	158.0	10,165
		*Artibeus jamaicensis*	WHU_Ajam_v2	202.0	2589
		*Desmodus rotundus*	ASM294091v2	94	26,870

**Table 2:** Bat *TRIM* genes evolving under positive selection.

TRIM Gene	lnL (m7)	lnL (M8)	2ΔlnL	*p*-Value	Positive Selection Sites Identified by Both PAML and HYPHY
TRIM5	−32,644.82	−32,434.64	420.38	<0.001	7, 71, 93, 145, 162, 165, 179, 181, 188, 209, 230, 270, 283, 311, 324, 325, 326, 374, 378, 381, 383, 389, 401, 424, 433, 484, 490
TRIM13	−5282.01	−5250.82	62.37	<0.001	312, 346
TRIM20	−14,407.60	−14,385.42	44.37	<0.001	55, 183, 195, 225, 229, 255, 273, 371, 395, 671, 676, 677, 689
TRIM21	−5451.73	−5435.53	32.40	<0.001	46, 50, 60, 405
TRIM22	−21,774.11	−21,705.86	136.50	<0.001	4, 19, 45, 53, 89, 120, 146, 248, 290, 293, 302, 323, 353, 390, 404, 407, 409, 427, 453
TRIM23	−4970.69	−4964.70	11.97	0.003	561, 566
TRIM24	−11,079.17	−11,022.77	112.81	<0.001	3, 4, 6, 7, 21, 25
TRIM25	−10,989.77	−10,960.90	57.75	<0.001	93, 313, 337, 350, 412, 423, 429
TRIM29	−12,198.15	−12,190.60	15.09	0.001	770
TRIM33	−10,163.69	−10,159.81	7.76	0.021	5
TRIM34	−8042.75	−8035.51	14.48	0.001	9, 107, 163, 505
TRIM37	−10,419.03	−10,181.11	475.82	<0.001	972, 980
TRIM38	−19,320.11	−19,312.62	14.98	0.001	47, 181, 229
TRIM43	−8556.49	−8538.28	36.42	<0.001	50, 169, 283, 314, 332, 429, 432, 442
TRIM44	−7106.06	−7072.26	67.60	<0.001	6, 271, 272, 274
TRIM45	−8511.14	−8505.68	10.91	0.004	551, 580
TRIM54	−4339.02	−4326.17	25.71	<0.001	270
TRIM55	−8167.15	−8128.54	77.23	<0.001	169
TRIM56	−9273.58	−9266.46	14.24	0.001	1, 6, 10
TRIM60	−37,265.91	−37,192.27	147.27	<0.001	37, 100, 168, 234, 275, 308, 311, 320, 324, 354, 358, 375, 385, 386, 387, 394, 402, 458
TRIM65	−6766.77	−6763.43	6.68	0.036	34, 36, 364
TRIM69	−5868.04	−5856.62	22.85	<0.001	123, 202, 375
TRIM75	−29,293.72	−29,277.40	32.65	<0.001	134, 167, 462
TRIML2	−10,138.56	−10,125.39	26.33	<0.001	11, 14, 16, 26, 54, 101, 191, 335, 339, 438

**Table 3:** Transcriptomic data used for expression analysis of bat TRIM genes.

Species	Cell Type	Accession Number	Treatment
*Myotis lucifugus*	embryonic fibroblast cell	SRR18761563–SRR18761565	4 h_IFNa
		SRR18761566–SRR18761568	4 h_control
*Myotis daubentonii*	kidney cell line	SRR8062281–SRR8062283	6 h_control
		SRR8062284–SRR8062286	24 h_control
		SRR8062287–SRR8062289	6 h_IFNa
		SRR8062293–SRR8062296	6 h_RVFV
		SRR8062297–SRR8062299	24 h_RVFV

## Data Availability

Not applicable.

## Supplementary Materials

The following supporting information can be downloaded at https://www.mdpi.com/article/10.3390/v15040875/s1, Figure S1: Genomic representation of the *TRIM60/61/75* locus in bats and other selected mammals; Table S1: Accession number of bat TRIM family proteins; Table S2: Positive selected sites identified by PAML (M8), or HYPHY (SLAC, FEL, MEM, FUBAR).
