# Supplementary material for: Positive Selection and Duplication of Bat TRIM Family Proteins
Source: Viruses. 2023 Mar 29;15(4):875. doi: 10.3390/v15040875 (PMC10145180; doi:10.3390/v15040875)
Supplement: Supplementary file 1 [file viruses-15-00875-s001.zip › viruses-2275137-supplementary.pdf]

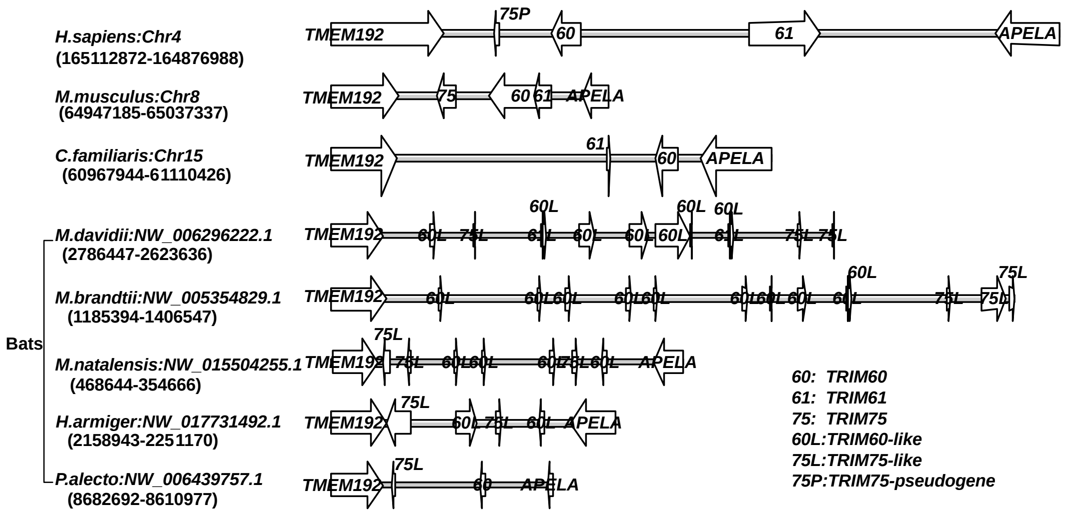

Figure S1. Genomic representation of the *TRIM60/61/75* locus in bats and other selected mammals. The common boundaries of the *TRIM60/61/75* locus are *TMEM192* and *APELA*. Genes are indicated by hollow arrows with size proportional to their length.

Table S1. Accession number of bat TRIM family proteins.

	A.jamaicensis	D.rotundus	E.fuscus	H.armiger	M.brandtii	M.davidii	M.lucifugus	M.molossus	M.myotis	M.natalensis	P.alecto	P.discolor	P.kuhlii	P.vampyrus	R.aegyptiacus	S.hondurensis
TRIML1	XP_036984802.1	XP_024418406.1	XP_028008276.1	XP_019490518.1	XP_005884904.1	XP_006753855.1	XP_006092704.1	XP_036106507.1	XP_036153945.1	XP_016064484.1	XP_006916547.1	XP_028382048.1	XP_036314569.1	XP_011363578.1	XP_015984823.2	XP_036910983.1
TRIML2	XP_036984801.1	XP_024418405.1	XP_008144485.1	XP_019483810.1	XP_005884910.1	XP_006753867.1	XP_006092751.1	XP_036106483.1	XP_036152968.1	XP_016064468.1	XP_006916546.2	XP_028382156.1	XP_036313040.1	XP_011363577.1	XP_036084204.1	XP_036910982.1
TRIM1<br>MID2	XP_037023007.1	XP_024407374.1	XP_027988151.1	XP_019518001.1	XP_005865280.1	XP_015420765.1	XP_006082318.1	XP_036127104.1	XP_036159915.1	XP_016053961.1	XP_024904660.1	XP_035872827.1	XP_036316594.1	XP_023389878.1	XP_036082307.1	XP_036921735.1
TRIM2	XP_037023967.1	XP_024424623.1	XP_028007658.1	XP_019496759.1	XP_005871486.1	XP_015423535.1	XP_006081861.1	XP_036096367.1	XP_036167696.1	XP_016053260.1	XP_024898412.1	XP_035887808.1	XP_036281298.1	XP_023385944.1	XP_036089453.1	XP_036914262.1
TRIM3	XP_036990406.1	XP_024425100.1	XP_008158069.1	XP_019488030.1	XP_014388821.1	XP_015423515.1	XP_006105633.1	XP_036098110.1	XP_036181134.1	XP_016072737.1	XP_006915722.1	XP_035886057.1	XP_036284168.1	XP_011383478.1	XP_036077004.1	XP_036907485.1
TRIM4	XP_036990813.1	XP_024427122.1	XP_028001573.1	XP_019488953.1				XP_036136639.1	XP_036165823.1	XP_016073176.1	XP_006918710.1	XP_028362446.1	XP_036265906.1	XP_011384617.1	XP_036082148.1	
TRIM5	XP_037009768.1<br>XP_037002205.1	XP_024430436.1<br>XP_024430433.1<br>XP_024430439.1<br>XP_024430426.1	XP_027993432.1<br>XP_008158694.1	XP_019523972.1	XP_005884915.2<br>XP_005867353.1<br>XP_014389423.1<br>XP_014397487.1<br>XP_014399628.1	XP_015422163.1<br>XP_006767986.2<br>XP_006767987.1<br>XP_006770130.1<br>XP_006752668.1	XP_014305207.1<br>XP_023614536.1<br>XP_023614537.1<br>XP_006094622.1<br>XP_014305203.1	XP_036115119.1<br>XP_036113725.1<br>XP_036115125.1<br>XP_036109537.1<br>XP_036115127.1	XP_036181107.1<br>XP_036181100.1<br>XP_036181111.1<br>XP_036181110.1<br>XP_036180353.1	XP_016070465.1<br>XP_016072768.1<br>XP_016070412.1		XP_035886047.1<br>XP_035886082.1	XP_036283986.1		XP_036077542.1	XP_036907464.1<br>XP_036885687.1<br>XP_036893640.1
TRIM6	XP_037002195.1	XP_024430445.1	XP_008158663.1	XP_019523991.1	XP_005860882.1		XP_006094617.1	XP_036115454.1	XP_036181101.1	XP_016070518.1	XP_006915740.1	XP_028371304.2	XP_036284005.1	XP_011381433.1	XP_015982322.1	XP_036893644.1
TRIM7	XP_037009346.1			XP_019490279.1			XP_014303339.2	XP_036095976.1	XP_036170035.1		XP_015453443.1	XP_035870841.1	XP_036306354.1		XP_016013889.2	XP_036908772.1
TRIM8	XP_037015304.1	XP_024411450.1	XP_008142471.1	XP_019491872.1	XP_005878304.1	XP_015414756.1		XP_036120951.1	XP_036192023.1	XP_016075566.1	XP_006920964.1	XP_028367172.1	XP_036293755.1	XP_011384124.1	XP_016010005.1	XP_036919932.1
TRIM9	XP_037011696.1	XP_024423856.1	XP_008137279.1	XP_019488788.1	XP_005867951.1	XP_006773839.1	XP_006100289.1	XP_036126825.1	XP_036162525.1	XP_016053591.1	XP_006920259.1	XP_028363760.1	XP_036292946.1	XP_011383863.1	XP_036095110.1	XP_036910179.1
TRIM10	XP_036997200.1	XP_024413569.1		XP_019488327.1	XP_014391303.1		XP_023600963.1	XP_036103798.1	XP_036212436.1	XP_016070256.1	XP_024903839.1	XP_035879633.1		XP_011382299.1	XP_015976150.1	XP_036901906.1
TRIM11	XP_036996374.1	XP_024416685.1	XP_028016269.1	XP_019490470.1<br>XP_019487974.1	XP_005864957.1			XP_036096962.1	XP_036168248.1	XP_016066113.1	XP_006923338.1	XP_028390380.1	XP_036306497.1	XP_011370226.1	XP_015983949.2	XP_036924816.1
TRIM13	XP_036990901.1<br>XP_037020080.1	XP_024425663.1<br>XP_024422552.1	XP_028017178.1	XP_019506395.1	XP_014392672.1	XP_015417797.1	XP_023604460.1	XP_036106342.1	XP_036154403.1	XP_016060955.1	XP_024900545.1	XP_028382429.1<br>XP_028382121.2	XP_036315116.1	XP_011368623.1	XP_015998575.1	XP_036917274.1<br>XP_036910991.1
TRIM14	XP_036996676.1	XP_024415735.1	XP_008140089.1	XP_019505070.1			XP_006096546.1	XP_036117456.1	XP_036186142.1		XP_006911607.1	XP_028380619.1	XP_036289136.1	XP_011364609.1	XP_016001770.2	XP_036891593.1
TRIM15				XP_019488325.1				XP_036103800.1			XP_006913708.3			XP_011382301.1	XP_015976122.2	
TRIM16	XP_037021458.1	XP_024420635.1	XP_027989679.1	XP_019497030.1	XP_005857393.1	XP_006762801.1	XP_006097430.1	XP_036124419.1	XP_036197596.1	XP_016051991.1	XP_006908465.1	XP_028390424.1	XP_036308061.1	XP_011357290.1	XP_015988000.2<br>XP_016016790.2	XP_036926833.1
TRIM17	XP_036996379.1	XP_024416643.1		XP_019487971.1		XP_015427035.1	XP_006100257.2	XP_036096964.1	XP_036168251.1	XP_016066126.1	XP_006923339.3	XP_035888697.1	XP_036306503.1	XP_011370225.2	XP_015983951.2	XP_036924819.1
TRIM18<br>Midline-1	XP_037013412.1	XP_024425656.1	XP_027988574.1	XP_019506773.1	XP_005880872.1	XP_006763866.1	XP_006104243.1	XP_036127395.1	XP_036160901.1	XP_016067119.1	XP_006906100.1	XP_035872532.1	XP_036276376.1	XP_011361206.1	XP_015995641.1	XP_036911466.1
TRIM19<br>PML	XP_036990409.1	XP_024412999.1	XP_008155295.1	XP_019523497.1	XP_005882857.1	XP_006753305.1	XP_006087912.1	XP_036113734.1	XP_036204422.1	XP_016057187.1	XP_024896162.1	XP_028363520.1	XP_036312022.1	XP_023380152.1	XP_015980459.2	XP_036902968.1
TRIM20	XP_037010557.1	XP_024409044.1	XP_028001472.1	XP_019488407.1	XP_014400628.1	XP_015414020.1	XP_023611040.1	XP_036136091.1	XP_036166422.1	XP_016066853.1	XP_006913958.1	XP_028366066.1	XP_036265728.1	XP_011374846.1	XP_015977766.2	XP_036899257.1
TRIM21	XP_036991629.1	XP_024428422.1	XP_027994367.1		XP_005885969.1	XP_006762400.1	XP_014316233.1	XP_036115934.1	XP_036181084.1	XP_016070523.1	XP_006905883.2	XP_035884805.1	XP_036283469.1	XP_011375746.1	XP_016013478.2	XP_036924534.1
TRIM22	XP_036995001.1<br>XP_037002200.1<br>XP_037002204.1	XP_024430440.1<br>XP_024430446.1	XP_027993442.1<br>XP_008158665.1	XP_019523971.1<br>XP_019523961.1	XP_014395497.1<br>XP_014390732.1	XP_015415099.1<br>XP_006770132.2	XP_023614551.1<br>XP_014316245.2	XP_036115126.1	XP_036181096.1<br>XP_036181102.1	XP_016070466.1	XP_024905226.1	XP_035886074.1<br>XP_035886065.1	XP_036284042.1<br>XP_036284002.1	XP_023380642.1<br>XP_023380643.1	XP_016005550.2<br>XP_036077538.1	XP_036907458.1<br>XP_036907454.1<br>XP_036891411.1
TRIM23	XP_037006667.1	XP_024434187.1	XP_008160093.1	XP_019507971.1	XP_005858001.1	XP_006762481.1	XP_006081467.1	XP_036135027.1	XP_036164033.1	XP_016058128.1	XP_006913142.1	XP_035876229.1	XP_036265159.1	XP_011369389.1	XP_015991713.2	XP_036918745.1

TRIM24<br>TIF1A	XP_037015148.1	XP_024410784.1	XP_008148712.1	XP_019508832.1	XP_014402186.1	XP_015428153.1	XP_023601075.1	XP_036102180.1	XP_036184910.1	XP_016077393.1	XP_024901618.1	XP_028381122.1	XP_036284009.1	XP_011357865.1	XP_016017357.2	XP_036905014.1
TRIM25	XP_037019619.1	XP_024429210.1	XP_008146137.1	XP_019492147.1	XP_014400311.1	XP_006758754.1	XP_006095695.1	XP_036123733.1	XP_036196376.1	XP_016067850.1<br>XP_016062677.1	XP_015454759.1	XP_028375735.1	XP_036310092.1	XP_023389286.1	XP_036077874.1	XP_036893322.1
TRIM26	XP_036997201.1	XP_024413562.1	XP_027989633.1	XP_019488333.1	XP_005871639.1	XP_015426142.1	XP_014301754.1	XP_036103795.1	XP_036212416.1	XP_016070260.1	XP_006913709.1	XP_035879625.1	XP_036284849.1	XP_011382297.1	XP_015976184.1	XP_036901904.1
TRIM27<br>RFP	XP_037008114.1	XP_024413671.1		XP_019490301.1				XP_036104931.1			XP_024900105.1	XP_028364838.1		XP_011380254.1	XP_015976191.1	XP_036901924.1
TRIM28<br>TIF1B	XP_037004326.1		XP_008159311.1	XP_019488847.1	XP_005857336.1<br>XP_014392028.1	XP_015416286.1	XP_023600474.1	XP_036129753.1	XP_036167295.1	XP_016071317.1	XP_015445219.1	XP_035869278.1	XP_036281936.1	XP_011382936.1	XP_015981020.2	XP_036885593.1<br>XP_036903480.1
TRIM29	XP_037017864.1	XP_024425342.1	XP_028014192.1	XP_019519113.1	XP_014394335.1	XP_015416111.1	XP_023604790.1	XP_036116040.1	XP_036167140.1	XP_016063144.1	XP_006915711.1	XP_035884283.1	XP_036286145.1	XP_023382015.1	XP_015974305.2	XP_036884431.1
TRIM32	XP_037010605.1	XP_024418187.1	XP_008150332.1	XP_019512391.1	XP_014384371.1	XP_015425380.1	XP_006091144.1	XP_036117713.1	XP_036185719.1	XP_016069241.1	XP_015448880.1	XP_028364934.1	XP_036288713.1	XP_011359263.1	XP_036094412.1	XP_036915044.1
TRIM33	XP_037008266.1	XP_024426356.1	XP_008145585.1	XP_019514739.1	XP_014390149.1	XP_015419019.1	XP_014314418.2	XP_036127929.1	XP_036198226.1	XP_016078775.1	XP_024908066.1	XP_028358497.1	XP_036308731.1	XP_023391318.1	XP_036090953.1	XP_036894825.1
TRIM34	XP_037002196.1	XP_024430435.1	XP_008158661.1	XP_019523990.1	XP_005860880.1	XP_006752662.1	XP_006094619.1	XP_036114173.1	XP_036181098.1	XP_016070520.1	XP_015447817.1<br>XP_015447843.1		XP_036283976.1	XP_011381436.1<br>XP_011381454.1	XP_036077535.1	XP_036893642.1
TRIM35	XP_036994989.1	XP_024424871.1	XP_028015206.1	XP_019491739.1	XP_014395533.1	XP_006764828.1	XP_006082013.1	XP_036096561.1	XP_036168604.1	XP_016070368.1	XP_006917034.1	XP_028376784.1	XP_036279685.1	XP_011358609.1	XP_016009788.1	XP_036898145.1
TRIM36	XP_037022961.1	XP_024419098.1	XP_028005529.1		XP_014399726.1	XP_015413941.1	XP_014317469.1	XP_036135435.1	XP_036165225.1	XP_016080430.1	XP_006913352.2	XP_035876559.1	XP_036265847.1	XP_011356548.1	XP_036081949.1	XP_036888225.1
TRIM37	XP_037019632.1	XP_024429057.1	XP_028010520.1	XP_019492252.1	XP_014385417.1	XP_006756252.1	XP_023615380.1	XP_036123513.1	XP_036196287.1	XP_016066146.1	XP_024897585.1	XP_035889545.1	XP_036296093.1	XP_023376161.1	XP_015978552.2	XP_036925928.1
TRIM38	XP_037008898.1	XP_024426053.1	XP_028009730.1	XP_019488748.1<br>XP_019488733.1<br>XP_019488741.1<br>XP_019488745.1<br>XP_019488728.1<br>XP_019488729.1	XP_014384083.1<br>XP_014392543.1<br>XP_014392561.1	XP_015423610.1<br>XP_015416973.1	XP_023602431.1<br>XP_023602432.1<br>XP_014306189.2	XP_036129231.1	XP_036199251.1<br>XP_036199250.1	XP_016071746.1	XP_006923551.1	XP_028359273.1	XP_036309071.1<br>XP_036309009.1	XP_023390166.1	XP_036090653.1	XP_036907307.1
TRIM39	XP_036997203.1	XP_024413566.1	XP_027989637.1	XP_019490250.1	XP_005863306.1	XP_006774096.1	XP_023600935.1	XP_036103796.1	XP_036212434.1	XP_016070263.1	XP_006913711.1	XP_028364454.1	XP_036284852.1	XP_011382296.1	XP_015976168.1	XP_036902775.1
TRIM40								XP_036104511.1		XP_016070246.1	XP_024903835.1	XP_028365945.1		XP_023381157.1	XP_036093744.1	
TRIM41	XP_037009295.1	XP_024408032.1	XP_027991236.1	XP_019490278.1	XP_005884574.1<br>XP_005862395.1	XP_006766918.1<br>XP_006761511.1	XP_006085719.1<br>XP_006109915.3	XP_036095970.1	XP_036169202.1<br>XP_036159989.1	XP_016057017.1	XP_015453491.1<br>XP_006921080.1	XP_035869931.1	XP_036306346.1	XP_023387590.1<br>XP_011364369.1	XP_016013859.2<br>XP_016019775.1	XP_036908767.1
TRIM42	XP_036995169.1	XP_024421876.1	XP_008150821.1	XP_019501502.1	XP_005870045.1	XP_006759895.1	XP_006083823.1	XP_036123140.1	XP_036187427.1	XP_016062538.1	XP_006907252.1	XP_028374765.1	XP_036286406.1	XP_011355754.1	XP_015997338.1	XP_036917054.1
TRIM43			XP_008147435.1	XP_019483846.1<br>XP_019483843.1	XP_005864899.1		XP_023601914.1	XP_036114211.1			XP_006926933.1		XP_036284544.1<br>XP_036284539.1	XP_011361605.1<br>XP_011361606.2	XP_016008662.2	
TRIM44	XP_036984115.1	XP_024414840.1	XP_028009426.1	XP_019495077.1	XP_005864567.1	XP_015414501.1	XP_006103800.2	XP_036115310.1	XP_036182069.1	XP_016061709.1	XP_015441886.1	XP_035885142.1	XP_036282989.1	XP_023384445.1	XP_015978706.2	XP_036896409.1
TRIM45	XP_036991035.1	XP_024426274.1	XP_008145445.1	XP_019516988.1	XP_005860006.1	XP_015418964.1	XP_006097551.1	XP_036128565.1	XP_036200102.1	XP_016078851.1	XP_015450957.1	XP_035871034.1	XP_036308586.1	XP_011364844.1	XP_016016382.1	XP_036894773.1
TRIM46	XP_036988579.1	XP_024426713.1	XP_008153933.1	XP_019518247.1	XP_014396133.1	XP_006775830.1	XP_006097910.2	XP_036128306.1	XP_036200557.1	XP_016077114.1	XP_006923598.1	XP_028359073.1	XP_036298692.1	XP_011364726.1	XP_015991118.1	XP_036906484.1
TRIM47	XP_037013937.1	XP_024409791.1	XP_027986425.1	XP_019499777.1<br>XP_019499764.1		XP_015426249.1	XP_014301297.1	XP_036124963.1	XP_036195890.1<br>XP_036197559.1		XP_006912462.1<br>XP_015445093.1	XP_028376647.1	XP_036267999.1<br>XP_036268416.1	XP_011360365.1	XP_015983193.2	XP_036921227.1
TRIM48									XP_036160014.1							
TRIM49			XP_028012837.1			XP_006766425.2							XP_036284545.1<br>XP_036284541.1<br>XP_036284547.1			
TRIM50	XP_037010645.1	XP_024427074.1	XP_008139693.1	XP_019502387.1	XP_005877257.1	XP_006773437.1	XP_023609170.1	XP_036135753.1	XP_036166198.1	XP_016054556.1	XP_006918621.1	XP_028372652.2	XP_036264890.1	XP_011369834.1	XP_016001968.2	XP_036910614.1
TRIM51			XP_028012838.1								XP_006926931.1		XP_036284548.1	XP_011361603.1	XP_016008664.2	

TRIM52			XP_008156905.2		XP_014391990.1	XP_006766919.1		XP_036095982.1	XP_036169201.1	XP_016057019.1	XP_015453492.1		XP_036306363.1	XP_023387599.1	XP_036079935.1	
TRIM54	XP_037002458.1	XP_024408714.1	XP_008136571.1	XP_019492369.1	XP_005875023.1	XP_006779579.1	XP_023619244.1	XP_036118882.1	XP_036190950.1	XP_016065713.1	XP_006910495.1	XP_028372684.2	XP_036290833.1	XP_011354785.1<br>XP_011383085.1	XP_015984510.1	XP_036922759.1
TRIM55	XP_037004832.1	XP_024428043.1	XP_028014596.1	XP_019488231.1	XP_005867988.2	XP_015422670.1	XP_006109310.1<br>XP_023611268.1	XP_036120175.1	XP_036194288.1	XP_016061902.1	XP_024896974.1	XP_035887060.1	XP_036292938.1	XP_023389039.1	XP_016008370.2	XP_036914536.1
TRIM56	XP_036995113.1	XP_024427350.1	XP_028001069.1	XP_019502454.1	XP_014405642.1	XP_006773472.2	XP_023602810.1	XP_036135203.1	XP_036165513.1	XP_016054458.1	XP_024907289.1	XP_035876659.1	XP_036266197.1	XP_011369902.1	XP_036081369.1	XP_036910759.1
TRIM58	XP_037021705.1	XP_024416413.1	XP_008149749.1	XP_019489162.1				XP_036095548.1	XP_036170073.1		XP_006923341.1	XP_035888699.1	XP_036306308.1	XP_011370221.1	XP_015983952.2	XP_036924748.1
TRIM59	XP_036982280.1	XP_024418993.1	XP_028002322.1	XP_019482109.1	XP_014393451.1	XP_006753876.2	XP_006102735.1	XP_036098207.1	XP_036202946.1	XP_016071215.1	XP_006908246.1	XP_035873508.1	XP_036303218.1	XP_011378004.1	XP_016004583.2	XP_036902533.1
TRIM60	XP_036996409.1<br>XP_036996412.1	XP_024425064.1<br>XP_024424499.1	XP_028007960.1<br>XP_008159979.1<br>XP_008144839.1	XP_019506122.1<br>XP_019506119.1	XP_005860614.2<br>XP_005860607.1<br>XP_005871396.1<br>XP_014405902.1<br>XP_005860605.1<br>XP_005860608.1<br>XP_014397352.1<br>XP_005860610.1<br>XP_005863053.2<br>XP_005857391.2<br>XP_005860609.1	XP_006778198.1<br>XP_006771143.1<br>XP_015424186.1<br>XP_006771132.2<br>XP_006771139.2<br>XP_006769185.2<br>XP_015424185.1	XP_006085519.1<br>XP_006082057.1<br>XP_006085375.1<br>XP_014312894.1<br>XP_023606267.1<br>XP_023611695.1<br>XP_023608651.1<br>XP_006093847.2<br>XP_014305550.1<br>XP_006082053.1<br>XP_006082056.1<br>XP_006082054.2	XP_036098006.1<br>XP_036137591.1<br>XP_036137494.1<br>XP_036138169.1<br>XP_036138069.1	XP_036169282.1<br>XP_036169336.1<br>XP_036181774.1<br>XP_036171394.1<br>XP_036189687.1<br>XP_036194081.1<br>XP_036169073.1<br>XP_036204235.1<br>XP_036169177.1<br>XP_036168754.1	XP_016053295.1<br>XP_016053282.1<br>XP_016053285.1<br>XP_016053283.1	XP_006927091.1	XP_028377047.1<br>XP_028376960.1	XP_036279954.1<br>XP_036282180.1<br>XP_036279950.1<br>XP_036281117.1<br>XP_036279951.1<br>XP_036280244.1<br>XP_036279955.1<br>XP_036314225.1<br>XP_036279952.1<br>XP_036279949.1<br>XP_036279953.1	XP_023388480.1	XP_016010054.2<br>XP_036089258.1	XP_036914221.1<br>XP_036914126.1
TRIM61						XP_015424189.1	XP_006082055.1		XP_036169906.1			XP_028377048.2				
TRIM62	XP_037005392.1	XP_024410307.1	XP_008155201.1	XP_019514581.1	XP_014404979.1	XP_006777415.1	XP_006094266.1	XP_036112057.1	XP_036205743.1		XP_015454257.1	XP_028369269.1	XP_036301158.1	XP_011356206.1	XP_036076492.1	XP_036917408.1
TRIM63	XP_036991675.1	XP_024410511.1	XP_028011076.1	XP_019505661.1	XP_005875323.2	XP_006777571.2	XP_014322865.1	XP_036112463.1	XP_036207050.1	XP_016071928.1	XP_006924459.1	XP_028368830.1	XP_036300673.1	XP_011355952.1	XP_015985314.1	XP_036888754.1
TRIM64	XP_037009482.1<br>XP_037017898.1<br>XP_036995583.1<br>XP_037017908.1			XP_019483848.1	XP_014400484.1	XP_006767198.1<br>XP_015421622.1					XP_006926769.1	XP_028370668.1	XP_036284537.1	XP_023388208.1	XP_016008660.2	XP_036898845.1
TRIM65	XP_037013884.1		XP_008153407.1	XP_019499745.1			XP_006109954.1	XP_036124896.1	XP_036196340.1			XP_028376425.1	XP_036268421.1		XP_036079121.1	XP_036921208.1
TRIM66	XP_036989749.1	XP_024414729.1	XP_028015698.1	XP_019484827.1	XP_014401327.1	XP_006763549.1	XP_023610357.1	XP_036114664.1	XP_036181180.1	XP_016062087.1	XP_024894573.1	XP_035884871.1	XP_036283123.1	XP_023378700.1	XP_036077483.1	XP_036906020.1
TRIM67	XP_036992664.1		XP_028013905.1	XP_019521378.1	XP_005856423.1			XP_036133550.1	XP_036210823.1			XP_028387130.1	XP_036292228.1		XP_036091474.1	XP_036916130.1
TRIM68	XP_036991624.1	XP_024428536.1	XP_008159458.1	XP_019489255.1	XP_014388566.1	XP_006762422.1	XP_014316235.1	XP_036115788.1	XP_036181087.1	XP_016070521.1	XP_024900016.1	XP_035884811.1	XP_036283013.1	XP_023379664.1	XP_016013480.2	XP_036924518.1
TRIM69		XP_024423637.1	XP_008141307.1	XP_019500419.1			XP_014305780.1	XP_036123644.1	XP_036155872.1	XP_016067413.1	XP_006920476.1	XP_028363377.1	XP_036299457.1	XP_011353470.1		
TRIM71	XP_037004115.1		XP_008152182.1	XP_019486275.1	XP_014403868.1		XP_014310283.1	XP_036121975.1	XP_036188760.1		XP_015452396.1	XP_028374094.1	XP_036286414.1	XP_011360586.1	XP_015996238.1	XP_036926649.1
TRIM72	XP_036986328.1	XP_024416349.1	XP_027983248.1	XP_019484054.1	XP_014385277.1	XP_015426163.1		XP_036136016.1	XP_036166097.1	XP_016073127.1	XP_006914229.1	XP_028378238.1<br>XP_028386087.2	XP_036268927.1	XP_011376890.1	XP_015988313.2	XP_036923130.1
TRIM75	XP_036996426.1<br>XP_036996413.1<br>XP_036996411.1	XP_024424498.1<br>XP_024424756.1<br>XP_024424502.1<br>XP_024424609.1	XP_008144795.1<br>XP_008144820.1<br>XP_008144850.1<br>XP_008144806.2	XP_019506146.1<br>XP_019506117.1	XP_005865783.1<br>XP_014390508.1<br>XP_005860869.1<br>XP_014390468.1<br>XP_005860616.1<br>XP_005860618.1<br>XP_014399511.1	XP_015427596.1<br>XP_015424192.1<br>XP_006771087.1	XP_014311692.1<br>XP_006082059.1<br>XP_023605499.1<br>XP_006082058.1<br>XP_006082060.1<br>XP_023608630.1<br>XP_006093533.1<br>XP_006100042.1<br>XP_006089747.1	XP_036097152.1<br>XP_036097403.1<br>XP_036138168.1	XP_036169865.1<br>XP_036170783.1<br>XP_036169904.1<br>XP_036169907.1<br>XP_036170785.1<br>XP_036154915.1<br>XP_036186344.1<br>XP_036206166.1	XP_016053296.1<br>XP_016053286.1<br>XP_016053281.1	XP_015448764.1	XP_028374879.1<br>XP_028377597.1<br>XP_028376764.1	XP_036280245.1<br>XP_036280701.1<br>XP_036280684.1<br>XP_036280700.1<br>XP_036280236.1	XP_011362038.1	XP_036089259.1	XP_036914128.1<br>XP_036914125.1<br>XP_036914223.1
TRIM77	XP_037017904.1	XP_024408183.1						XP_036113755.1			XP_006926932.1	XP_028370265.1		XP_011361604.1	XP_016008661.2	

Table S2. Positive selected sites identified by PAML(M8), or HYPHY (SLAC, FEL, MEM, FUBAR).

Gene	PAML					HYPHY			
	lnL M7	LnL M8	2ΔlnL (M7 vs. M8)	p_value (M7 vs. M8)	M8 [a]	SLAC [b]	FEL [c]	MEME [d]	FUBAR [e]
TRIM5	-32644.82	-32434.64	420.38	0.000	7,46,48,49,61,71,79,93,98,122,145,148,152,155,161,162,165,174,176,179,180,181,185,188,191,195,209,210,211,213,226,230,234,244,269,270,283,288,297,306,307,308,309,310,311,314,317,319,320,324,325,326,327,328,329,330,331,332,333,334,335,336,337,338,339,340,342,343,349,363,365,366,368,373,374,376,378,379,380,381,382,383,385,386,387,388,389,390,391,392,393,394,395,396,397,398,401,402,410,412,413,414,421,422,423,424,425,426,427,428,429,432,433,438,442,476,484,485,486,487,489,490,491,493,494,495	162,209,268,270,311,325,344,424,433,480	4,7,71,73,93,97,149,156,162,165,181,188,209,223,230,268,270,275,283,311,313,321,324,325,326,344,360,409,416,424,433,478,480	4,9,18,23,29,54,71,73,97,108,138,145,149,156,162,165,179,181,188,202,209,222,223,246,251,252,256,270,275,276,283,311,313,324,325,326,344,359,360,374,378,381,383,389,401,403,409,411,416,424,433,456,467,478,480,484,490	7,162,188,209,270,311,324,325,326,344,424,433,480
TRIM13	-5282.01	-5250.82	62.37	0.000	1,2,4,312,346,412		312,346	59,389	36,133,312,346
TRIM20	-14407.60	-14385.42	44.37	0.000	55,59,65,104,174,183,193,195,217,225,229,255,273,302,310,371,395,425,431,432,577,661,670,671,675,676,677,678,680,681,684,687,688,689	55	55,218,229,395	55,58,68,106,108,115,119,122,137,180,183,194,195,200,203,214,218,223,225,230,238,255,263,264,267,273,281,282,289,313,320,322,351,354,361,363,364,371,378,386,387,389,400,437,445,504,511,526,533,564,569,630,655,660,663,665,669,671,673,674,676,677,679,689	55
TRIM21	-5451.73	-5435.53	32.40	0.000	46,50,60,405,416		46,50,60,180,260,405	46,50,60,125,180,260,360,405	46,50,60,180, 260,405
TRIM22	-21774.11	-21705.86	136.50	0.000	4,5,10,19,23,44,45,46,47,48,49,53,79,86,87,89,92,120,121,122,146,189,198,228,248,253,258,260,262,269,288,290,293,297,302,303,323,338,340,344,346,348,353,390,392,403,404,406,407,409,412,427,428,453,510	19,290,409	7,19,53,67,68,91,151,290,293,302,353,357,390,404,407,409,427,471	4,7,13,19,29,37,41,45,50,62,67,68,74,105,110,120,137,145,146,151,187,217,239,248,275,276,279,290,293,298,302,315,323,330,353,357,390,402,404,409,449,451,493,502	19,53,89,248,302,400,407,409,427,453
TRIM23	-4970.69	-4964.70	11.97	0.003	11,23,555,559,560,561,562,566			561,566	
TRIM24	-11079.17	-11022.77	112.81	0.000	3,4,5,6,7,8,9,13,14,16,17,19,20,21,22,25,47,48,52,100,103,105,106,107,112,115,116,117,118,119,120,121,122,123,124			3,4,6,7,21,25,46,54,56,58,59,63,64,355,385,516,615,735,747	25
TRIM25	-10989.77	-10960.90	57.75	0.000	54,72,89,93,149,281,313,316,337,341,350,354,356,368,371,387,389, 390,391,392,397,407,412,423,428,429,478,588,589,615,623		412,423	93,313,347,350,412,423,429,440,530	337,412
TRIM29	-12198.15	-12190.60	15.09	0.001	140,142,770		770	29,447,472,622,724,761,770,782,786	770
TRIM33	-10163.69	-10159.81	7.76	0.021	5,6			2,3,5,7	7,710
TRIM34	-8042.75	-8035.51	14.48	0.001	9,107,159,163,168,283,366,414,505,509		9,107,136,189	55,81,88,133,136,163,189,205,218,303,357	9,107,163,189,505
TRIM37	-10419.03	-10181.11	475.82	0.000	968,969,970,971,972,973,974,975,976,977,979,980,981,982,983,985, 986,987,988,989,990			7,768,972,980	980
TRIM38	-19320.11	-19312.62	14.98	0.001	47,50,55,56,181,206,228,229	229	27,89,104,148,229	4,8,9,27,28,46,47,52,53,57,59,71,76,81,85,89,92,94,96,104,107,127,148,152,157,168,181,189,190,205,214,227,229	27,89,229

TRIM43	-8556.49	-8538.28	36.42	0.000	4,8,50,68,82,106,112,137,169,172,173,184,205,272,283,289,292,314,323,332,361,385,429,430,432,440,442,445,455,462		50,332	50,177,181,186,229,237,283,314,429,458	50,169,314,332,432,442,456
TRIM44	-7106.06	-7072.26	67.60	0.000	2,6,10,21,25,59,260,266,267,269,270,271,272,274			5,6,26,31,38,42,48,50,182,271,272,273,274	
TRIM45	-8511.14	-8505.68	10.91	0.004	19,24,77,84,163,359,450,501,506,535,546,548,549,551,568,570,571,573,574,577,579,580			262,283,400,497,498,504,505,515,518,525,540,544,551,552,560,580	304,580
TRIM54	-4339.02	-4326.17	25.71	0.000	270				270
TRIM55	-8167.15	-8128.54	77.23	0.000	169			57,58,63,67,80,90,93,94,97,102,113,169	
TRIM56	-9273.58	-9266.46	14.24	0.001	1,3,6,10,83,259,353,415,420,421,442,476			1,6,7,8,10,13,252,294,479,505	
TRIM60	-37265.91	-37192.27	147.27	0.000	4,37,84,100,125,149,163,168,169,195,197,202,234,275,290,308,311,317,320,321,322,323,324,325,330,354,356,358,360,373,375,384,385,386,387,388,389,390,392,393,394,395,396,397,398,399,400,401,402,429,444,452,454,458	37,274,277,311,320,324,354,372	18,37,110,238,240,245,274,277,311,320,324,354,372,386,446	2,3,18,37,42,48,83,95,100,107,110,127,141,150,153,156,168,176,188,198,234,237,238,240,245,274,275,277,295,301,306,308,311,315,320,324,332,350,352,354,358,369,372,375,385,387,394,402,414,416,420,443,446,450,455,457,458	18,37,274,277,311,320,324,354
TRIM65	-6766.77	-6763.43	6.68	0.036	32,34,35,36,38,40,84,148,149,172,179,181,182,195,209,216,220,230,245,247,248,249,267,276,280,341,364			7,8,11,12,15,26,30,36,41,42,45,73,75,76,80,86,88,96,97,101,105,108,115,118,120,125,130,137,138,140,141,160,166,183,184,188,205,211,217,226,232,256,266,289,294,296,299,300,302,305,343,362,363,364	34,36
TRIM69	-5868.04	-5856.62	22.85	0.000	36,37,49,123,199,200,201,202,209,215,219,221,225,226,251,282,290,334,357,358,363,373,375		123	24,34,123,202,228,354,361,364,367,371,375	123
TRIM75	-29293.72	-29277.40	32.65	0.000	47,59,90,134,167,170,180,182,197,269,270,328,381,386,397,454,462,464	38,85	38,85,95,134,154,159,167,215,229,291,457	9,13,21,24,38,73,83,84,85,87,95,98,101,111,121,139,154,159,167,209,215,225,229,236,240,243,273,288,291,329,377,380,402,405,412,418,420,429,435,457,462	38,215
TRIML2	-10138.56	-10125.39	26.33	0.000	2,4,5,8,9,10,11,12,13,14,15,16,17,18,26,32,48,54,55,59,77,88,101,136,137,147,148,150,152,154,170,177,191,195,198,209,210,222,224,232,270,291,324,335,339,357,425,437,438		26,54,184,335,337,339	1,11,14,16,26,54,101,158,163,184,189,191,230,289,335,438,439	

a) Codons with posterior probabilities >0.9 in the BEB analyses under M8 model.
b) Codons with significance level <0.05
c) Codons with significance level <0.05
d) Codons with significance level <0.05
e) Codons with posterior probabilities >0.9
